# Navigating toxicity in lung cancer immunotherapy: challenges and advances in Nano medicine drug delivery

**DOI:** 10.1186/s43046-026-00358-7

**Published:** 2026-06-29

**Authors:** Rishab Jain, Ananda Kumar Chettupalli, Sarad Pawar Naik Bukke, Shikha Yadav, Vidya Sankarapandian, Ungo-Kore Hussain Yahaya

**Affiliations:** 1 Galgotias University, Greater Noida, India; 2 Adamas University, Kolkata, India; 3 Kampala International University, Western Campus, Ishaka - Bushenyi, Uganda; 4 Kampala International University, Kampala, Uganda

**Keywords:** Lung cancer, Immunotherapy, Nano medicine, Toxicity, Immune checkpoint inhibitors, Targeted drug delivery, Nano carriers

## Abstract

Lung cancer is still among the most malignant cancers, with immunotherapy becoming a ground-breaking treatment option. The ICIs and other immunotherapeutic drugs usually cause severe immune-related adverse effects curtailing their therapeutic efficacy. Meeting this challenge, Nano medicine-based drug delivery systems have gained considerable interest as they hold the promise of increasing therapeutic benefits at the same time as reducing toxicity. This chapter discusses the complex balance between the effectiveness and toxicity of lung cancer immunotherapy, underlining the application of nanotechnology in maximizing drug delivery. Nano carriers like liposomes, polymeric nanoparticles, dendrites, and lipid-based systems have demonstrated the capacity to augment the bioavailability of pharmaceuticals, facilitating tumour-specific environments, and alleviating systemic side effects. Other options suggest that stimuli-responsive and ligand-functionalized Nano platforms can provide spatial control over the immune response by enhancing infiltration to the tumour site while reducing toxicity to healthy organs. On the battlegrounds of nanomedicine, inhibiting resistance mechanisms consolidated with immune checkpoint inhibitors and conventional chemotherapeutics has conferred better therapeutic responses upon the patient. Its stability, bio-distribution, and regulatory pathway concerns still challenge clinical translation. The chapter discusses recent advances in preclinical and clinical testing and describes the development of Nano medicine-based regulatory T-cell-directed immunotherapy for lung and lung-associated cancers. The problems concerning both toxicity and the application of nanotechnology for targeting therapy will open new avenues toward developing safer and more effective antitumor immunotherapeutic regimes for lung cancer.

## Introduction

Lung cancer remains a major cancer threat worldwide because it ranks among the most frequently occurring and lethal types of cancer. The World Health Organization reports that lung cancer leads to approximately 1.8 million deaths every year, which makes it the primary cause of cancer deaths worldwide. The disease is classified into two main types: NSCLC, which constitutes about 85% of cases, and SCLC, which is more aggressive and makes up the remaining 15%. NSCLC may be subdivided into squamous cell carcinoma and large cell carcinoma. Every subclass, is defined by molecularly precise genetic profile to target, could be classified into a myriad of subsets. Metastatic lung cancer survival rates, encompassing both NSCLC and SCLC variants, are drastically reduced, having a survival rate of around 4% after 5 years [1]. SCLC is an extremely metastatic and treatment-resistant neoplasm. While comprehensive global data on SCLC is insufficient, it is predicted that SCLC is responsible for approximately 15% of lung cancers and leads to over 200,000 deaths each year. People suffering from regional SCLC confined to lung may get surgical resection of the primary tumour, associated with improved survival rates. About 70% of individuals was found to have SCLC have metastatic illness, commonly presenting with macro-metastases in the skeleton, the brain, the liver, and the lymph nodes. Surgical resection in these people has demonstrated inferior efficacy compared to radiation or chemotherapy and is hence infrequently executed [2].

Immune checkpoint pathways, including PD-1/PD-L1 and CTLA-4, are exploited by cancer cells to evade immune surveillance. ICIs augment autoimmune the immune response to cancer cells by blocking the production of the designated checkpoint antibodies. Four ICIs have been given the green light by the FDA in the United States for NSCLC: atezolizumab, durvalumab, pembrolizumab, and nivolumab. However, the latter two aim against anti-PD-L1, the former two focus on the PD-1 receptor. Comparing pembrolizumab monotherapy to platinum-based chemotherapy, many phase III randomised controlled trials show that the former significantly improves survival rates in patients with advanced NSCLC, while also decreasing the risk of adverse events of grade 3 or above. Pembrolizumab monotherapy or atezolizumab monotherapy is the conventional first-line treatment for NSCLC patients with a PD-L1 tumour percentage score less than 1% [3]. Despite these significant clinical benefits, immunotherapy is associated with important limitations, including variable patient response, resistance mechanisms, and the development of irAEs. These toxicities arise due to non-specific immune activation and can affect multiple organ systems, including the lungs, gastrointestinal tract, endocrine glands, skin, and liver. Among these, pneumonitis is particularly concerning in lung cancer patients due to pre-existing pulmonary compromise. Patients with incurable stage III cancers may benefit from durvalumab monotherapy, according to the PACIFIC study in NSCLC. Based on recent clinical research, the first line of treatment for advanced non-small cell lung cancer is a combination of chemotherapy and immune checkpoint inhibitors. patients yield improved survival rates and diminished side effects. However, immunologically connected side effects caused by immunological checkpoint inhibitors exceed those associated with chemotherapy. The principal aetiology of adverse events has a significant influence on the endocrine glands, gastrointestinal tract, skin, lungs, liver, cardiovascular system, neurological system, and blood because of autoreactive T cell activation caused by the blocking of immunological checkpoint receptors PD-1/PD-L1 and CTLA-4. Consequently, Therapeutic regimens in clinical practice must encompass the treatment and management of adverse effects. Nevertheless, the ideal PD-1/L1 therapy remains not clear. The influence of PD-L1 on immunotherapy’s efficacy in individuals is still debated. Thus, a network meta-analysis was conducted in our study to assess first-line therapeutic approaches and determine the optimal treatment option [4].

One explanation for cancer’s persistence is the wide variety of mutations that cancer cells may undergo. This diversity allows cancer cells to evolve resistance to conventional treatment methods. As a group, lung cancer and its main histologic subtypes include predictably only one out of five tumour forms exhibiting the highest prevalence of somatic mutations. An expanding variety of therapeutic methodologies concentrates on T cells for investigative objectives. T-cell-based immunotherapy has come to be seen as a crucial component of cancer treatment. Nevertheless, the elimination of cancer the immune system’s handling of cells is an intricate procedure that necessitates several conditions. Initially, tumour-associated antigens from dying cells are released into the tumour microenvironment., where they are subsequently collected by antigen-presenting cells (APCs) [5].

Antigen-loaded APCs subsequently process and present antigens in conjunction with MHC molecules to the cell surface and transport them to lymphoid organs. Naive T cells in lymphoid organs identify specific peptide-MHC complexes via the T cell receptor, starting the process of effector T cell priming and activation. The result is that cancer cells infect healthy tissues via the bloodstream when separated effector T cells leave lymphoid organs. Through direct or indirect immunological pathways, T cells destroy cancer cells after recognising them with peptide-MHC complexes that interact with T cell receptor to match antigens. Nivolumab, an ICI that interferes with PD-1 expression, was the first of its kind to be developed and used as the first-line therapy for those with severe NSCLC [6]. For individuals whose platinum-based chemotherapy-treated advanced squamous or non-squamous non-small cell lung cancer has progressed, randomised phase III trials showed that nivolumab had a higher OS and objective response rate compared to docetaxel. Pembrolizumab and atezolizumab, two PD-1 and PD-L1 inhibitors, were subsequently authorised by the US FDA.

These drugs were for the same purpose because they outperformed docetaxel in the second line. At the beginning of therapy for advanced NSCLC, ICIs were used because of their effectiveness in the second-line scenario. Immune checkpoint inhibitors, either alone or in addition to chemotherapy that uses platinum, have been shown to have long-lasting effects and much improved results of phase III clinical trials for overall survival compared to chemotherapy alone, greatly expanding the pool of potential patients whose non-small cell lung cancer has progressed to an advanced stage with EGFR mutations or ALK translocations. The rise of immune-evasion mechanisms is a major issue. Lung cancer patients who undergo immunotherapy demonstrate two types of resistance pathways which include both innate and acquired resistance mechanisms. Primary resistance occurs when tumours show no response to ICIs from the start because they have a low TMB and insufficient neoantigen presentation and an immunosuppressive TME. The process of acquired resistance develops over time because it depends on two factors: genetic changes and variations in how immune cells enter the body and how the immune system learns to protect itself [7].

The development of effective treatment methods needs to address the challenge of immune checkpoint inhibitors which produce toxic side effects and treatment resistance because these inhibitors have shown better clinical results. Nanomaterials’ capacity to positively alter the pharmacokinetic properties of chemotherapeutic drugs and relieve, to some extent, the therapeutic dosage limitations caused by systemic toxicity is one of their key advantages. Furthermore, nanoparticles could transport drugs to lung tissue in a sustainable manner, which reduces the frequency of doses and improves patient compliance. The administration of anticancer medications specifically to tumours areas has been made possible with the development of numerous nanocarriers in recent years. These include biomimetic nanoparticles, polymers, liposomes, and inorganic nanoparticles. Nano carriers can be functionalised for targeting, environmental responsiveness, imaging, and other uses after being loaded with drugs by electrostatic interactions, hydrophobic contacts, or covalent bonding. Nanomaterials have the potential to improve inhaled delivery for lung cancer in addition to systemic therapy. Because of their large drug-loading capacity, nanoparticles have the potential to enable the co-delivery of many medications. For systemic drug administration, PEGylation is a common modification for many nanoparticles; it improves biocompatibility, stability, and RES clearance evasion. Exogenous UV thermal response, glutathione response, hypoxia response, and pH reaction are all examples of TME reactions that can be designed to improve nanomaterial functionality [8].

Despite the clinical success of ICIs, their use is frequently limited by irAEs, which can affect multiple organ systems, including the lungs, liver, skin, and endocrine glands. These toxicities arise from systemic immune activation and remain a major barrier to optimal therapeutic outcomes. Therefore, there is an urgent need for strategies that can enhance the therapeutic index of immunotherapy by minimizing off-target effects while preserving antitumor efficacy. Nanomedicine-based drug delivery systems show potential to reduce toxicity through their ability to deliver drugs directly to specific targets while maintaining controlled release and controlling immune system functions [9].

### Toxicity in lung cancer immunotherapy [10]

Immunotherapy combined with ICIs has established a new standard for lung cancer treatment through its significant enhancements of patient survival rates and their medical results. The treatment of cancer, particularly lung cancer, benefits from immunotherapy which provides multiple advantages yet carries dangers through its potential to cause irAE disorders. These harmful effects might influence numerous bodily systems, resulting in serious problems and, in some instances, life-threatening illnesses. Comprehending the causes, constraints, and obstacles of immunotherapy-associated toxicity is crucial for formulating safer and more effective treatment methods for lung cancer [11].

**Fig. 1:**
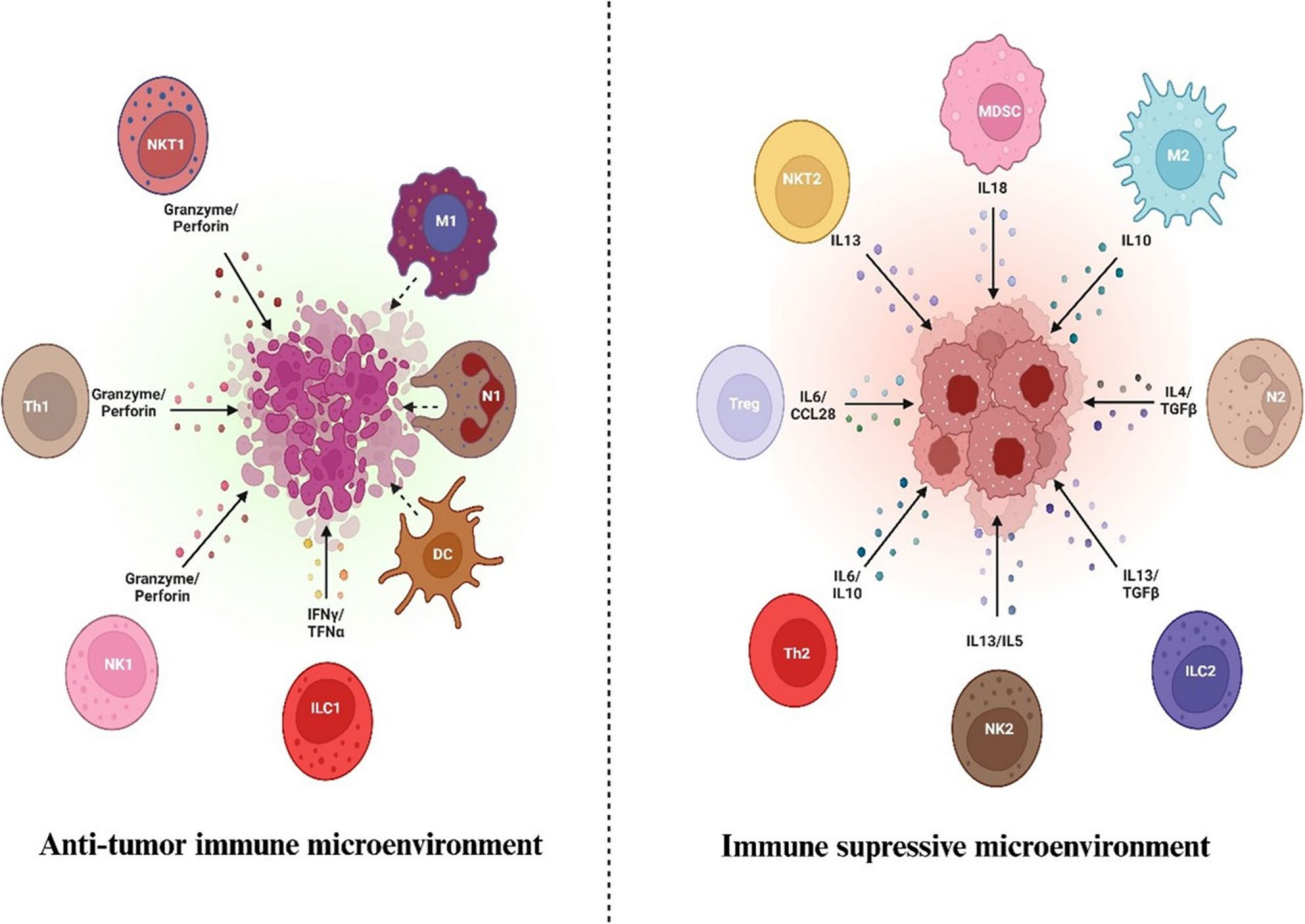
Tumour Microenvironment and Immune Checkpoint Interactions in Lung Cancer

Immunological checkpoint molecules are negative regulators that are crucial for maintaining immunological homeostasis and attenuating immune responses to avert autoimmunity. Nonetheless, tumour cells may manipulate this mechanism to elude the host immune response by suppressing immunological response by activating immune system checkpoints. Potential therapeutic benefits for cancer patients may arise from immunological activation, enhanced antitumour immunity, and the interruption of inhibitory signals brought about by blocking immune checkpoint pathways. Multiple ICIs have recently received FDA approval as a means of dealing with various cancers. Biological agents such as Ipilimumab, Cemiplimab, Nivolumab, Pembrolizumab, and Dostarlimab are antibodies that are designed to specifically target substances. Other examples of such agents are Atezolizumab, Avelumab, and Durvalumab, including Relatlimab, LAG-3, and lymphocyte activation gene [12]. Activated T cells are the ones that primarily express CD152, also known as CTLA-4, a CD28 homolog that is continuously expressed on regulatory T cells. The ligands CD80 and CD86 are most often found on surface APCs. Starting an immune response that is both inhibitive and stimulatory, respectively, is possible when they attach to CTLA-4 or CD28. Although CD28 and CTLA-4 both bind to CD80 and CD86, CTLA-4 binds with significantly more affinity. Consequently, it competes for interactions with T cells, which in turn lowers their activation of CD80 and CD86 with the co-stimulatory protein CD28 [13]. CTLA-4 may limit CD28-mediated stimulatory signalling, which in turn dampens immunity against relatively weak antigens, such as those found in the body or tumours. To further maintain self-tolerance, Treg cells with intrinsic CTLA-4 expression reduce immunological responses by reducing cytokine release and effector T cell proliferation. Lethal multiorgan tissue damage, severe T cell-mediated autoimmunity, and widespread lymphoproliferation may result from CTLA-4’s absence [14] (Fig. 1).

In addition to activated T cells, B cells, and natural killer cells, some myeloid cells also express CD279. The promotion of peripheral tolerance occurs via interactions with APCs, which include macrophages, dendritic cells, and even certain B cells. Immune overactivation may cause serious host tissue damage due to immune-mediated mechanisms if PD-1 signalling is not present. Although they share 38% identity, the expression patterns of PD-L1 and PD-L2 are different [15]. When it comes to the immune response, PD-L1 and PD-L2 have distinct functions, like IFN and IL-17 considerably upregulate PD-L1 expression in APCs, while IL-4 enhances PD-L2 expression. More so, some cancer cells and immune cells that infiltrate tumours may express PD-1 and PD-L1, which helps to establish a microenvironment that suppresses the immune system where cancer cells are able to evade the body’s natural defences against tumours [16].

Many different types of immune cells, such as activated T cells, T regulatory cells, B cells, dendritic cells, and natural killer cells, express LAG-3 (CD223), a member of a new family of immunological checkpoint molecules. LAG-3 and its ligand may interact reduce T cell activity and enhance Treg cell activation, two mechanisms that contribute to immune system homeostasis. Major histocompatibility complex II (MHC II), a ligand for CD4, is the primary ligand for LAG-3. Because LAG-3 binds to MHC-II much more strongly than CD4, it might potentially compete with CD4 for the ability to decrease T cell activation [17]. It has been found in the literature that there are several possible ligands for LAG-3. Hepatic sinusoid endothelial cell lectin, fibrinogen-like protein 1, and galactose lectin-3 are among them. The TME contains many ligands on various cell types; when these ligands interact with LAG-3, it is possible that tumour immune evasion is promoted by reducing anti-cancer immune responses [18].

Despite the increasing importance of immunotherapy, especially in lung cancer, many patients do not benefit much from medicines that suppress the PD-1/PD-L1 checkpoint. In addition, some patients have toxicities connected to the immune system, which may have a negative impact on their quality of life escalating healthcare expenses and leading to significant morbidity or mortality. Therefore, it is essential to develop clinically relevant predictive biomarkers to accurately identify individuals who are most likely to benefit and to exclude those with little chances of effectiveness and/or heightened risk of harm [19].

### PD-L1 expression

When it comes to metastatic NSCLC, the National Comprehensive Cancer Network recommends using just PD-L1 as a biomarker, in addition to genetic driver mutations. In stage IV NSCLC, this biomarker has shown predictive potential in many trials. Most research points to PD-L1 expression levels as a potential predictor of immunotherapy efficacy and as a tool for patient selection. But several studies have cast doubt on PD-L1’s validity as a biomarker for gauging how well PD-1/PD-L1 checkpoint treatments will work [20]. It is becoming increasingly apparent that PD-L1 expression in non-metastatic conditions is important for prognosis. In the PACIFIC trial, individuals treated with durvalumab who tested negative for PD-L1 had worse progression-free survival (PFS) and overall survival than patients treated with durvalumab who tested positive for PD-L1 [21]. There was no difference in outcomes between PD-L1-positive and negative patients, according to a correlative investigation of a smaller phase II trial using consolidation pembrolizumab after chemoradiation. Atezolizumab enhanced disease-free survival in the adjuvant scenario after surgery and adjuvant chemotherapy only in the PD-L1 ≥ 1% cohort, with the greatest effect shown in the PD-L1 ≥ 50% subgroup [22].

### Tumour mutational burden

TMB Counting all mutations per megabase in the exotic regions of the tested genes in a cancer sample is called the Cancer Mutational Burden. The number of neoantigens and newly transcribed proteins is directly proportional to the mutation frequency. One theory is that a higher concentration of neoantigens makes tumours more immunogenic and increases the chance that patients may react positively to checkpoint inhibition. Multiple studies in tumour-agnostic and lung cancer have shown the usefulness of tissue and blood-based TMB to predict the efficacy of different ICIs [23]. The KEYNOTE-158 study’s findings supported the FDA’s recent approval of pembrolizumab for patients with tumours of any kind showing a high tumour mutational load (≥ 10 mut/Mb). There is evidence that the predictive power of composite biomarkers that combine PD-L1 and TMB is higher. There are currently no clinically meaningful markers that can be used to guide the administration of immune checkpoint inhibitors to patients with advanced SCLC [24]. Although SCLC often shows an increased tumour mutational load and immune-mediated paraneoplastic syndromes, it usually does not benefit from checkpoint inhibition as much as NSCLC. Regardless of PD-L1 subgroup, clinical trials evaluating the efficiency of ICI in combination with chemotherapy as a first-line treatment for extensive-stage SCLC found no statistically significant differences in response rates or clinical effectiveness of ICI [25].

### Biomarker-guided nanomedicine for toxicity reduction

While PD-L1 expression and TMB are widely recognized as predictive biomarkers for immunotherapy response, their integration with nanomedicine strategies offers a promising approach for reducing irAEs [26]. The design of nanocarriers based on biomarkers allows for accurate delivery of immunotherapeutic drugs which reduces both systemic drug exposure and unintended immune system activation. The use of PD-L1-targeting ligands on nanoparticles leads to improved tumor-specific accumulation which enables precise immune checkpoint control while decreasing systemic adverse effects. The delivery of siRNA through nanocarriers to target PD-L1 expression enables precise control of immune activities within the tumor microenvironment. The two methods together for actual use different from laboratory testing lead to better treatment results while controlling excessive immune response because this type of immune reaction causes irAEs [27].

Patients with high TMB who have greater neoantigen load will respond better to immunotherapy treatment. They will gain advantages from enhanced dosing methods which work together with nanocarrier systems. The system provides sustained controlled release of immunotherapeutic agents through nanoparticles. This system lowers peak systemic concentrations which decreases toxicity risks while sustaining treatment effectiveness. Recent studies have shown that combining biomarker-based patient selection with advanced nanocarriers can create customized immunotherapy treatments. The strategies enable better patient identification which leads to customized treatment methods that maintain safety while delivering effective results. The research shows two fields of study which can work together to design better treatments for lung cancer through biomarker science and nanomedicine [28].

Cancer cells differ from normal cells due to changes in their DNA or proteins. Consequently, they could spread more easily and multiply faster. Taking advantage of these differences, targeted cancer treatment zeroes in on certain proteins and genes that have a role in cancer development and progression. It limits damage to healthy cells by inhibiting the development and propagation of cancer cells. Due to their unique side effect profiles, targeted therapies are frequently employed in the treatment of advanced lung cancers after conventional treatments have failed. Many drugs have been developed to target these pathways, and there are many targeted drugs that the Food and Drug Administration has approved. Inhibitors that target There are now available EGFR, ALK, PI3K/AKT/mTOR, RAS-MAPK, RET, MET, BRAF, and NTRK/ROS1 variants, together with PD1 and CTLA4 molecules, are accessible for NSCLC, and several of them have become standard treatment options for certain individuals [29]. Multiple studies have shown the efficacy of small-molecule inhibitors targeting Vascular Endothelial Growth Factor and its receptors (VEGF/VEGFR) in the treatment of different malignancies, including NSCLC. There are two FDA-approved anti-VEGF agents for advanced NSCLC: Both ramucirumab (Cyramza/anti-VEGFR antibody) and Avastin (anti-VEGF antibody) are used as monotherapy either alone or in conjunction with chemo. Angiogenesis relies on the VEGF route, which is the subject of extensive research. Since VEGF causes tumour-related immunosuppression, new evidence suggests its significance in cancer aetiology may be more substantial than first believed [30].

Researchers looked at how miRNAs regulated the VEFG pathway in a few studies. Effects on NSCLC rat models include decreased miR-199a messenger RNA expression and increased VEGF. According to their claims, miR-199a limits NSCLC cell proliferation by targeting and reducing the activity of the signalling route of hypoxia-inducible factor 1-alpha/ VEGF. Recent findings shown that in lung cancer cells, miR-214 targeted the Growth Family Member 4 inhibitor, which in turn activated the HIF-1α pathway and resulted in increased levels of Matrix Metallopeptidase 2 and VEGF. According to these results, additional research into the targeted inhibition of the VEFG pathway through a different method is necessary [31]. A protein involved in cellular signalling pathways that govern cell division, maturation, and growth is encoded by gene. Finding effective treatments for human cancers is made more difficult by the fact that KRAS is the oncogene most frequently mutated. Consequently, despite significant efforts aimed at developing medications to inhibit KRAS or its signalling pathways, KRAS has generally been regarded as undruggable. Nonetheless, some recent studies demonstrate promising outcomes, heralding a new and exciting age. Like sotorasib, another KRAS G12C (OFF) inhibitor called adagrasib (MRTX849) stabilises the switch II pocket It forms an inactive conformation with GDP attached to it, which it binds to KRAS G12C irreversibly. Dagrazib has shown anticancer effectiveness against brain metastases in clinical studies, and it can pass the blood-brain barrier. Individuals with advanced or metastatic malignancies, such as NSCLC with the KRASG12C mutation, and a history of therapy for these conditions were eligible to participate in the phase I and II adagrasib studies. Several combination treatments utilising adagrasib are now in development, and phase III research (KRYSTAL-12) is comparing adagrasib and docetaxel in those who have just had treatment for NSCLC with a KRASG12C mutation [32]. Initiating several signalling pathways that contribute to cell development, differentiation, proliferation, and survival is the role of the EGFR, also known as ErbB1 or HER1. When ligands bind to the EGFR tyrosine kinase, the receptor is activated by homo- or heterodimerization and auto-phosphorylation of tyrosine-rich areas in the cytoplasm. Key intermediary pathways downstream are active, including the PIK3CA/AKT1/mTOR and RAS/RAF1/MAP2K1/MAPK1 kinases. Additionally, they can stop ligands from attaching to the outside of the cell in addition to producing endocytosed and destroyed antibody-receptor complexes [33].

The four monoclonal antibodies that target EGFR at present are matuzumab, cetuximab, necitumumab, and panitumumab. For the treatment of advanced non-small cell lung cancer, two phase III trials, FLEX and BMS099, used cetuximab in conjunction with platinum doublet chemotherapy. When compared to conventional chemotherapy, EGFR-TKIs significantly enhance overall response rate, progression-free survival, and quality of life with very few adverse effects. Thanks to EGFR-TKIs, a new era of precision medicine and tailored treatment has begun, and NSCLC management has shown tremendous improvements [34].

PD-1/PD-L1 and CTLA-4 inhibitors used in ICIs boost antitumor immune response, but they also produce serious toxic effects because their universal operational mechanism drives total body immune system activation. The pathways need to be understood because they affect both the success of therapeutics and the development of methods to minimize immune-related side effects. The research on PD-L1 expression and TMB biomarkers now extends beyond their ability to forecast treatment outcomes because scientists investigate these markers for their potential to show which patients will experience immune system side effects, thus enabling doctors to create tailored and safer immunotherapy plans [35].

### Integration of biomarkers with nanomedicine for toxicity mitigation

The combination of predictive biomarkers which include PD-L1 expression and TMB together with nanomedicine delivery systems creates a new method that improves treatment accuracy while minimizing irAEs. The primary function of PD-L1 and TMB assessments involves predicting how patients will respond to treatment yet researchers now discover their value for developing safe immunotherapy solutions. Biomarker-based patient classification identifies candidates who will gain from immune checkpoint inhibitors because it protects nonresponsive patients from harmful treatment effects. The spatial delivery method of immunotherapeutic agents which targets tumor sites allows nanomedicine to improve this system [36].

Researchers have created nanoparticles which target PD-L1 and antibody-conjugated nanocarriers to deliver immune checkpoint inhibitors directly to PD-L1-expressing tumour cells which results in decreased systemic immune activation and related toxic effects. The redox-sensitive and siRNA-loaded nanoparticles which target PD-L1 expression have proven their capability to achieve local checkpoint pathway inhibition while maintaining minimal off-target impacts. The nanocarrier-based controlled release systems enable patients with high tumour mutational burden to achieve optimal immune activation which helps to avoid excessive systemic immune responses that lead to immune-related adverse effects. Nanomedicine-based co-delivery strategies which include immune adjuvants or modulators show potential to improve therapeutic effectiveness in low-TMB tumours without requiring increased systemic doses [37].

Most studies which investigate these synergistic strategies have not progressed beyond their initial preclinical testing phase and they lack clinical validation. The standardization of these methods faces difficulties because different biomarkers and tumor types exhibit variable expression patterns. The combination of biomarker-guided therapy with nanomedicine development represents an important advancement toward personalized lung cancer treatment that detects treatment-related harmful effects.

### Nanomedicine-based drug delivery approaches

More than eighty five percent of lung cancer cases are NSCLC, and lung cancer in general remains a leading cause of cancer-related mortality worldwide. The introduction of immunotherapy through ICIs has established a new standard for lung cancer treatment because it enhances the body’s capacity to identify and destroy cancerous tumors. The implementation of the system faces multiple difficulties which include irAEs and drug resistance and inefficient drug administration. The medical field requires nanomedicine because it delivers advanced drug delivery solutions which enhance treatment outcomes while minimizing harmful effects to the entire body [38]. Nanoparticles, liposomes, dendrimers, and hybrid nanocarriers provide controlled drug release, increased bioavailability, and targeted delivery to tumour sites. Immunomodulatory nanoparticles can activate immune cells and modify the tumour microenvironment to enhance anticancer immunity. Cancer nanomedicine, in conjunction with immunotherapies, has the potential to enhance cancer susceptibility to immunotherapeutic treatments and suppress anti-tumor immune responses [39].

The primary limitation of cancer immune therapeutics is the effective delivery of oncogenes to the immune system. Small, microscopic particles systems encompass various forms, with the most popular platform for cancer immunotherapy, which has received FDA clearance, is nanoparticles made of polymers. Biodegradability, biocompatibility, and non-toxicity are some of the advantageous features shown by several polymers. These include chitosan, polyethylene glycol, and poly(lactide-o-glycolic) acid [40]. As a possible immunotherapeutic strategy, blocking the PD-1/PD-L1 pathway has been considered; however, this intervention has a poor response rate and leads to low cytotoxic T cell infiltration. A new kind of mesoporous nanocarrier with vastly porous silica around an inside core of upconverting nanoparticles to leverage an immunosuppressive microenvironment for addressing challenging tumour microenvironments [41]. The Lewis murine lung cancer cell line and a mouse model were used for evaluation after the nanocarrier was simultaneously loaded with photosensitiser molecules, the AL-9 peptide vaccine from IDO, and PD-L1 inhibitors. Engineered UCMS Pep-aPDL1 components may enhance local and systemic anti-tumor immunity by facilitating the infiltration of effector T-cells, boosting the elimination of IDO-expressing cancer cells, and triggering immunogenic cell death [42] (Table 1).

**Table 1:** Nanocarrier Platforms for Drug Delivery in Lung Cancer Immunotherapy

S. No	Nanocarrier Type	Key Advantages	Challenges	Examples of Use in Lung Cancer	Ref
1	Liposomes	High biocompatibility, ability to encapsulate diverse drugs, reduced systemic toxicity	Stability issues, rapid clearance, limited drug loading in some cases	Delivery of chemotherapeutics and immune checkpoint inhibitors	[43]
2	Polymeric Nanoparticles	Controlled and sustained release, tuneable properties, improved drug stability	Complex synthesis, potential immunogenicity	Delivery of immunomodulators and combination therapies	[44]
3	Dendrimers	High drug-loading capacity, precise structural control	Cytotoxicity concerns, accumulation in non-target tissues	Cytotoxicity concerns, accumulation in non-target tissues	[45]
4	Inorganic Nanoparticles (Gold, Silica, Quantum Dots)	Theranostic capabilities (imaging + therapy), enhanced tumor penetration	Long-term toxicity, poor biodegradability	Imaging-guided drug delivery and photothermal therapy	[46]
5	Hybrid Nanocarriers (Lipid-Polymer, Gold-Liposome)	Combined advantages of multiple systems, improved stability and targeting	High production cost, complex fabrication	Co-delivery of chemotherapeutic and immunotherapeutic agents	[47]

About half of the NSCLC patients with active KRAS mutations had a missing or deactivated STK11 gene (LKB1), which codes for serine/threonine kinase 11. Anti-PD-L1 and PD-1 immunotherapy is often ineffective against KRAS mutant NSCLC metastatic tumours. A humanised mouse model to demonstrate that carboplatin and pembrolizumab marginally and temporarily limit tumour development, but TUSC2, a tumour suppressor gene administered by nanovesicles effectively eliminates tumour growth in most subjects. TUSC2 influences a pro-immune microenvironment in tumours, increasing the sensitivity of KRAS/LKB1 tumours to carboplatin in combination with pembrolizumab. The restoration of TUSC2 was demonstrated to reduce mouse models of KRAS mutant lung cancer to improve the effectiveness of anti-PD-1 treatment and to increase PD-L1 expression [48]. In the early phases of clinical research, toll-like receptor (TLR) agonists have garnered interest as possible anticancer medicines due to their ability to modulate adaptive immunity and induce innate immunological responses. CpG oligodeoxynucleotides trigger a series of immunological responses, both innate and adaptive, by acting as agonists for Toll-like receptor 9. When administered locally to tumours, CpG enhances anticancer effects and activates dendritic cells, which in turn triggers a cascade of secondary effects including T-cell proliferation, activation of natural killer cells, and the generation of pro-inflammatory cytokines [49]. Endogenous microRNAs (miRs) play a key role in regulating both the adaptive and innate immune responses. Several microRNAs, including miR-155, miR-125, and miR-29, may cause tumour-associated macrophages to switch from an M2 phenotype that promotes tumour growth to an M1 phenotype that fights tumours and inflammation. Macrophages express more miR-125b than other immune cells, and this expression regulates macrophage activation. By enhancing cancer cell cytotoxicity or indirectly lowering their proliferation, overexpressing miR-125b in M1 macrophages with a viral vector boosts anti-tumour activity in a subcutaneous EL4 tumour model. Utilising nanoparticles based on hyaluronic acid-poly (ethylenimine) (HA-PEI) that encapsulate miR-125b to target CD44 [50]. Combination therapy demonstrates considerable potential in cancer treatment. Yang et al. have created hyaluronic acid-cisplatin/polystyrene-Poly metformin dual-prodrug co-assembled nanoparticles as a combination therapy for lung cancer, effectively addressing the asynchronous pharmacokinetic challenges associated with the free drug forms of cisplatin and metformin. The results of both in vitro and in vivo tests on Lewis’s lung cancer cells using this nano-platform show significant improvements in tumour accumulation and proliferation inhibition, apoptosis induction, CD4^+^ and CD8^+^ T cell elevation, TNF-α and IFN-γ cytokine production, and overall life extension in mouse models due to its powerful immunotherapeutic properties [51]. An innovative method of drug administration by combining magnetic iron oxide nanoparticles, fucoidan, and dextran (Dex) that has been functionalised with aldehyde, with T-cell activators and checkpoint inhibitors. They used an anti-PD-L1 antibody as the ICI and made it even better by adding an anti-CD3/CD28 antibody to activate the immune system. Subjects treated with IO-FuDex3-nano-biohybrid immunotherapy for 4T1-metastatic lung cancer had a longer median survival rate, fewer metastatic nodules, less systemic toxicity, and increased infiltration of CD4^+^ and CD8^+^ T cells [52]. Capabilities of AuPG nanoparticles, which are structures produced from polyaniline and include gold. When used in conjunction with PD-1 treatment, AuPG nanoparticles showed significant M1 macrophage polarisation, enhanced cytotoxic T cell response, tumour reduction, and increased production of immunogenic cytokines in lung cancer immunotherapy. By delivering the TP53 tumour suppressor gene to cancer cells, SGT-53, an active targeted nanoparticle, restores the functioning p53 protein and enhances anti-PD-1 immunotherapy. SGT-53 is a well-established medication for lung cancer that has successfully passed several rounds of clinical testing [53].

Nanomedicine has arisen as a viable strategy in lung cancer immunotherapy, with inorganic and hybrid nanocarriers significantly boosting drug transport, augmenting bioavailability, and reducing systemic toxicity. Inorganic nanocarriers include metallic nanoparticles, mesoporous silica nanoparticles, and quantum dots with unique physicochemical properties for controlled drug release, imaging-guided treatment, and increased targeting to tumours. These nanoparticles feature exceptional stability, large surface area, and the ability to be functionalized by ligands or antibodies for targeted therapy [54]. Among the numerous applications in immunotherapy, the studies of gold nanoparticles have largely concentrated on their ability to enhance the immune response by acting as carriers of immune checkpoint inhibitors and adjuvants to improve therapy efficacy. The syn-delivery of chemotherapy and immune-modulating agents through mesoporous silica nanoparticles enables sustained release of therapeutic agents which decreases their harmful effects on the whole body. The additional benefit of using inorganic nanocarriers stems from their capacity to conduct theragnostic operations through their built-in medical treatment and diagnostic examination capabilities which function on a single nanoplatform. Fluorescent imaging serves as an imaging technique which enables researchers to monitor drug delivery and tumor response and treatment effectiveness in real time. The tunable optical properties of quantum dots enable their usage in both bioimaging and customized drug delivery systems [55]. The clinical use of inorganic nanocarriers remains hindered by two major challenges which include their long-term biocompatibility assessment and their potential toxic effects. Hybrid nanocarriers combine the benefits of both organic and inorganic nanomaterials to address the limitations found in single-component nanocarrier systems. The hybrid systems establish biocompatibility through their organic components which include lipids and polymers while they maintain stability and imaging capabilities through their inorganic materials [56]. Lipid-polymer hybrid nanoparticles provide optimal lung cancer immunotherapy treatment because they achieve high drug encapsulation efficiency while maintaining longer circulation times and controlled drug release capabilities. Gold-liposome hybrid nanoparticles serve as delivery systems which transport both immunotherapy and photothermal therapy components to target cancer treatment. The system uses magnetic hybrid nanoparticles which respond to external magnetic fields for drug delivery purposes. The system enables researchers to achieve precise tumor targeting while the system reduces unwanted side effects, which leads to better treatment results. The hybrid nanoparticles that respond to environmental stimuli by releasing drugs according to specific tumor microenvironment parameters of pH and enzyme activity and redox potential show substantial potential to improve lung cancer treatment delivery [57].

The main benefit of nanomedicine drug delivery systems for drug delivery exists because these systems reduce the harmful effects that come from immunotherapy treatments. The passive and active targeting methods which enable tumour-specific accumulation of nanocarriers decrease their potential to activate immune systems outside the targeted area. The controlled and stimuli-responsive drug release system achieves its goal by enabling immunotherapeutic agents to be activated specifically in the tumour microenvironment which protects healthy tissues from exposure and decreases the chances of immune-related adverse events [58]. The three immune checkpoint inhibitors nivolumab, pembrolizumab, and atezolizumab provide significant clinical advantages to patients, yet their treatment effectiveness varies between different patient groups. The primary advantage of these agents comes from their ability to create lasting treatment results, yet only a small fraction of patients experience extended treatment success. The treatment response of tumors becomes restricted by primary resistance mechanisms and acquired resistance mechanisms which include both low tumor immunogenicity and adaptive immune evasion. The treatment establishes a connection to immune-related adverse events whose severity and pattern of occurrence remain unpredictable. The clinical results between NSCLC and SCLC show distinct outcomes because SCLC displays lower treatment response rates despite high tumor mutational burden which demonstrates the unique challenges of this disease [59].

### Nanomedicine strategies for mitigating immunotherapy-associated toxicity

Nanomedicine provides different methods to decrease immunotherapy treatment side effects. Targeted nanocarriers equipped with ligands or antibodies enable specific delivery of immune checkpoint inhibitors to tumor sites which results in decreased bodywide immune response. The tumor microenvironment can achieve targeted drug delivery through the use of pH and redox and enzyme-sensitive nanoparticles which create dual responsive systems for drug release [60].

Nanocarriers support dose optimization together with sustained drug release because they minimize toxic peak systemic concentrations. The co-delivery systems which combine immunomodulators with anti-inflammatory agents assist in maintaining immune activation while decreasing toxicity. The new techniques which use biomimetic nanoparticles and cell membrane-coated systems improve immune compatibility while decreasing unintentional immune reactions. The combination of these methods demonstrates how nanomedicine can help lung cancer immunotherapy become safer while maintaining or increasing treatment effectiveness [61].

### Targeted and stimuli-responsive nanocarriers

**Fig. 2:**
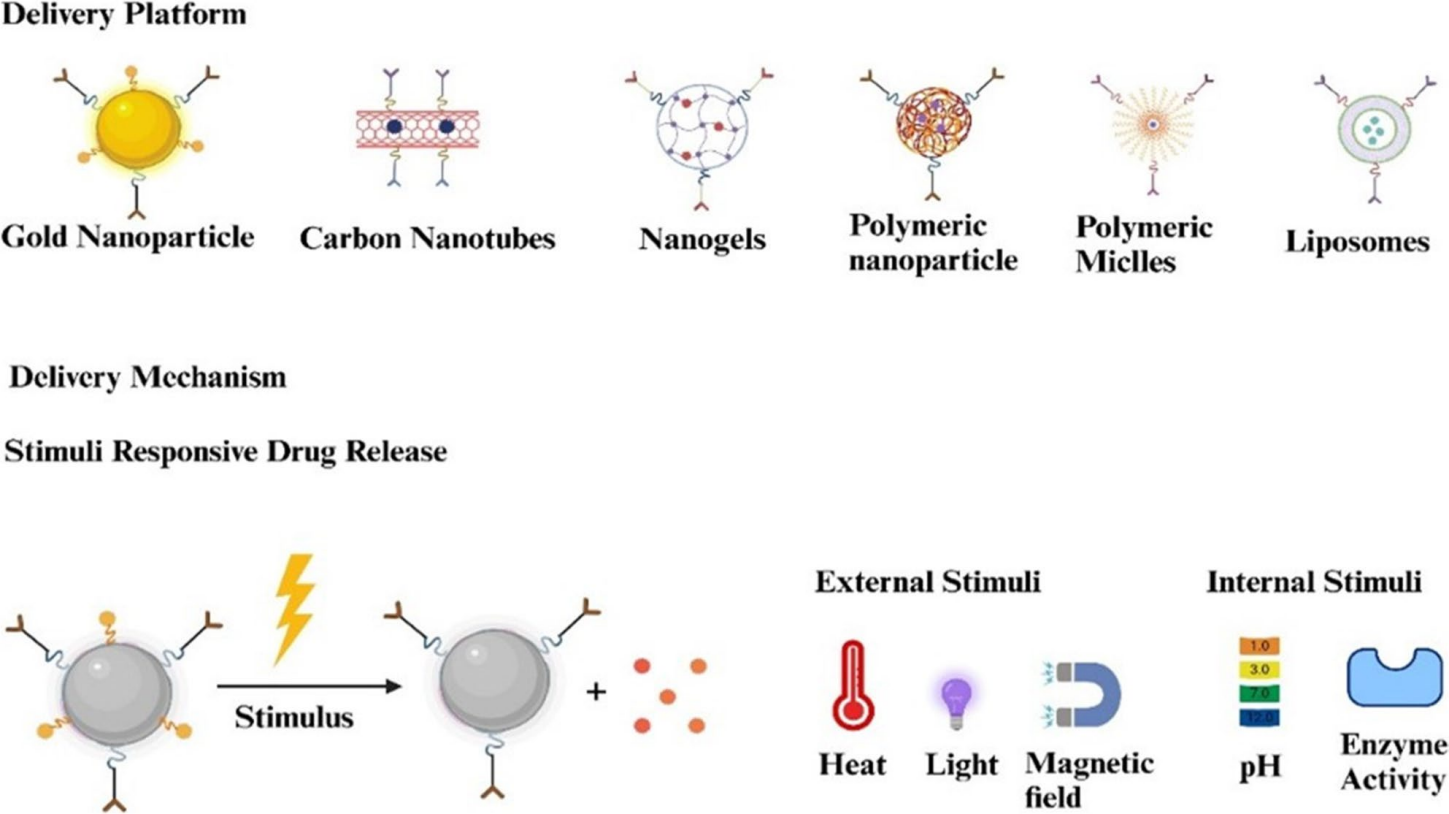
Stimuli-Responsive Nanocarriers for Targeted Drug Delivery

Lung cancer remains one of the most difficult tumours to manage because it exhibits aggressive behaviour and spreads rapidly while showing resistance to typical treatment methods. Immunotherapy, which uses ICIs as its main treatment approach, has revolutionized lung cancer therapy by increasing the body’s ability to fight against cancerous tumors. The need for more accurate drug delivery methods arises from the problems which include immune-related side effects and non-specific drug effects plus the inconsistent patient treatment outcomes. The introduction of targeted and stimuli-responsive nano carriers provides a solution to these challenges by enabling controlled delivery of medications to specific locations in the body. The nanocarriers were designed to respond to specific body signals which include pH levels and enzymatic processes and temperature changes to deliver cancer treatments directly at tumor sites while minimizing harmful effects to the rest of the body thus creating an effective treatment method for lung cancer [62]. Lung cancer worldwide, immunotherapy is still a major factor in the mortality rate from cancer and considerable advances in therapeutic modalities against it. However, it is the inherent variability in the tumour microenvironment that poses challenges associated with some forms of drug delivery, particularly because conventional therapies still demonstrate limited penetration into tumours and cause off-target damage. A variety of distinctive physiological conditions such as TMEs which are characterized by hypoxia, acidic pH, an increase in ROS, and overexpression of certain enzymes will allow personalized, stimuli-responsive drug delivery [63] (Fig. 2). Tumour microenvironment-specific Nano carriers promise to increase the precision with which immunotherapy is being developed in lung cancer and to maximize therapeutic benefits while reducing the systemic toxicity associated with treatment. A fundamental characteristic of the TME is its acidic pH, which arises from heightened glycolytic metabolism in neoplastic cells (the Warburg effect) [64]. The pH disparity (6.5–6.8 in tumours compared to 7.4 in normal tissues) has been used in pH-responsive medication delivery devices. Nanoparticles engineered with acid-sensitive polymers, PLGA and poly (β-amino esters) are materials that display structural changes when they encounter the acidic environment of tumors which leads to controlled drug release. The liposomal formulations which contain immune checkpoint inhibitors anti-PD-1 and anti-CTLA-4 antibodies have been created to deliver their contents exclusively at the acidic tumor microenvironment which helps to reduce immune-related adverse effects [65]. Researchers have developed pH-responsive micelles which deliver tumour-associated antigen peptides that boost immunotherapy antigen presentation and T cell activation in lung cancer treatment. The tumor microenvironment exhibits a defining characteristic which shows increased oxidative stress through its elevated levels of reactive oxygen species together with glutathione (GSH) components. The redox-responsive nanoparticles employ disulfide bonds which enable them to release drugs from their structure when excessive GSH levels exist in the cells [66]. The method allows for controlled delivery of immunomodulatory medications because it enables specific time-based delivery of active components which need protection against early breakdown. The research found that PD-L1 siRNA redox-sensitive liposomes created a strong immune pathway resistance against cancer in lung cancer research. The study showed that ROS-sensitive nanocarriers could deliver interleukin-12 and other pro-inflammatory cytokines while demonstrating improved T-cell movement and tumor shrinkage in animal tests [67].

The enzymatic activity in the TME facilitates enzyme-responsive drug delivery systems. MMPs, which are upregulated in lung cancer, may destroy extracellular matrix constituents and promote tumour invasion. Nanoparticles sensitive to MMP have been engineered with enzyme-cleavable peptide linkers that release medicines selectively in MMP-abundant settings. MMP-sensitive dendrimers containing ICIs have shown superior tumour penetration and increased treatment effectiveness in lung cancer mice. A further investigation revealed that MMP-responsive polymeric micelles delivering immune-stimulating chemicals markedly improved dendritic cell activation and antigen presentation, resulting in strong anti-tumour immunity. Hypoxia, a defining characteristic of solid tumours, significantly contributes to immune evasion and therapeutic resistance. Hypoxia-responsive drug delivery systems use tumour-specific oxygen gradients to activate medication release [68]. Hypoxia-activated prodrugs, including tirapazamine, have been integrated into nanoparticles to selectively release cytotoxic compounds in low oxygen environments, therefore minimising off-target damage.

Moreover, hypoxia-responsive liposomes containing T-cell agonists have shown the ability to augment T-cell infiltration and activation inside lung cancer tumours. Oxygen-releasing nanoparticles have been engineered to alter the hypoxic tumour microenvironment, therefore enhancing the effectiveness of radiation and immunotherapy. Recent improvements in multi-responsive nanocarriers have integrated pH, redox, and enzyme-sensitive methods to provide precise medication release in the tumour microenvironment [69]. For instance, dual-responsive nanoparticles that dissolve in acidic and high-glutathione milieus have been found to target lung cancer models more effectively. Additionally, multi-responsive liposomes delivering a combination of immune checkpoint inhibitors with chemotherapy have mutual advantages by blocking resistance mechanisms and enhancing treatment outcomes. This intelligent nano-carrier holds great promise for improving lung cancer immunotherapy with a controlled release of drugs designed for the tumour microenvironment. Solving these problems using ligand-functionalized and antibody-conjugated nanoparticles would allow more precise tumour targeting, improved therapeutic efficacy, and decreased systemic toxicity [70].

These nanocarriers exploit the unique molecular signatures of lung cancer cells and the TME to stimulate drug accumulation at the tumour site, limiting off-target effects. The ligand-functionalized nanoparticles use biomolecules, such as folic acid, transferrin, hyaluronic acid, and peptides, for binding to overexpressed receptors specifically on cancer cells, thus promoting receptor-mediated endocytosis and efficient drug delivery. Folic acid-functionalized nanoparticles have been designed to target the folate receptor, which is lung cancer cells often overexpress leading to improved delivery of chemotherapy and immunotherapy agents. Transferrin-conjugated nanoparticles capitalize on the elevated iron demand of the rapidly dividing lung cancer cells to facilitate the targeted delivery of drugs and ameliorate therapy. For monoclonal antibodies conjugated onto nanoparticles, enhanced specificity is conferred by binding to tumour-associated antigens or immune checkpoints. A common approach is involves combining nanoparticles with antibodies that target certain immune checkpoints, such as PD-L1 or PD-1, to suppress them locally at the cancer site [71]. This study indicates that PD-L1-conjugated polymeric nanoparticles enhance immune activation through higher local drug concentration and lower systemic exposure, thus mitigating immune-related side effects like pneumonitis and colitis. Besides ICIs, antibody-functionalized nanoparticles have been used for the delivery of chemotherapeutics, siRNA and cytokines that work in tandem to reprogram the immunosuppressive TME towards enhanced anti-tumour immunity. Nanoparticles targeting EGFR show enhanced treatment outcomes for NSCLC patients harbouring EGFR mutations by thereby selectively inhibiting tumor proliferation while sparing normal tissues. A significant benefit of ligand-functionalized and antibody-conjugated nanoparticles is their capacity to augment combination therapy [72]. Nanocarriers that are dual-functionalized to transport both immune checkpoint inhibitors and chemotherapeutic drugs have synergistic benefits, effectively bypassing resistance mechanisms and enhancing patient responses. Research has established that delivering antibody-conjugated nanoparticles carrying both PD-1 blockers and paclitaxel results in superior tumour reduction compared to single-agent treatments. The development of multifunctional nanoparticles which combine ligands and antibodies with stimuli-responsive elements enables more precise drug delivery through its ability to detect changes in pH and enzyme activity and redox status inside the tumour microenvironment. The advanced nanocarriers show great potential to improve clinical applications but face multiple challenges which include difficulties in large-scale production and maintaining stability and obtaining regulatory approval [73].

The development of controlled release mechanisms for lung cancer immunotherapy through nanomedicine enables precise drug delivery which maintains continuous treatment while minimizing both systemic side effects and immune-related adverse events. Conventional lung cancer therapies, including chemotherapy and ICIs, suffer from problems associated with rapid systemic clearance, off-target effects, and severely limited tumor penetration [74]. Toward this end, controlled drug release mechanisms have been devised using stimuli-responsive nanocarriers that discharge their payload in response to tumour-specific physiological conditions, including pH, enzyme activity, redox potential and external stimuli such as temperature, magnetic fields, and ultrasound. A common method of controlled delivery is pH-dependent drug delivery, which takes advantage of the tumour’s acidic media (pH 6.5–6.8) in contrast to normal physiological settings (pH 7.4). Acid-sensitive nanocarriers such as pH-responsive liposomes, micelles, and polymeric nanoparticles are designed to have acid-degradable linkers that activate release within the tumour microenvironment [75]. For example, pH-sensitive liposomes loaded with immune checkpoint inhibitors like anti-PD-L1 antibodies promote tumour development while reducing immune-related damage. Likewise, pH-responsive polymeric micelles have also been designed to selectively deliver immune-modulating agents into tumor tissues, where they would enhance T-cell activation and, consequently, the immune response. Another highly effective technique is made by enzyme-responsive nanocarriers designed to be responsive to tumour-associated enzymes, among them MMPs, cathepsins and proteases, to regulate drug release. MMP-sensitive nanoparticles use peptide-based linkers that are cleaved by MMPs for drug activation being site-restricted at the tumour location. MMP-responsive dendrimers containing checkpoint inhibitors have shown increased infiltration into lung tumours and higher effectiveness in immunotherapy. Moreover, enzyme-activated nanocarriers co-loaded with chemotherapeutic agents and immune checkpoint inhibitors have been engineered to facilitate sequential drug release, therefore efficiently surmounting tumour resistance mechanisms [76].

Redox-responsive medication release is a significant controlled release mechanism that utilises the elevated concentrations of ROS and glutathione (GSH) present in tumours. Redox-sensitive nanocarriers include disulfide bonds that are broken in high-GSH conditions, facilitating rapid intracellular drug release. Research indicates that redox-sensitive liposomes containing PD-1 inhibitors provide enhanced tumour targeting and diminish systemic immunosuppression. This polymeric micelle can be ROS responsive and, as such, can dose immunostimulatory drugs for an enforced anti-tumour immune response. Besides endogenously initiated mechanisms, temperature-sensitive nanocarriers are examples of external stimuli-responsive strategies with superb control of drug activation. The thermally sensitive liposomes containing immune checkpoint inhibitors release the medicines at very high temperatures (> 40 °C), which can be brought about by hyperthermia or radiofrequency ablation outside [77]. Thus, this should give rise to an increased specific concentration of immunotherapeutic agents in the tumour microenvironment while decreasing their systemic exposure. The use of external magnetic fields in controlling the drug-loaded nanoparticles targets tumour tissues, facilitating a targeted delivery. This application has been extended to lung cancer treatment such that the ICI bioavailability is increased while also activating T cells. Researchers are investigating ultrasound-responsive nanocarriers because they can release drugs in a controlled manner when exposed to focused ultrasonic sound. The use of immune checkpoint inhibitors and cytokines through ultrasound-sensitive liposomes and polymeric nanoparticles resulted in better immune system activation because they increased tumor penetration. The research tested three types of pH, enzyme and redox sensitive nanocarriers which reacted to outside forces and their controlled drug release showed significant accuracy improvements [78].

The clinical applicability of different nanocarrier systems depends on their unique strengths and weaknesses. Liposomes provide a biocompatible delivery system which can carry both hydrophilic and hydrophobic drugs yet they face problems with maintaining stability and experiencing fast body elimination. The synthesis process of polymeric nanoparticles requires complex procedures which create challenges while these nanoparticles provide controlled delivery. Dendrimers enable high drug loading and precise structural control yet their use faces problems because of their toxicity and ability to build up in non-target areas. The theragnostic capabilities of gold and silica-based inorganic nanoparticles enable medical use yet researchers must study their long-term body compatibility and elimination from the system. Hybrid nanocarriers face manufacturing and regulatory obstacles because their development increases system complexity and operational requirements (Table 2).

**Table 2:** Nanocarrier-based immunotherapy studies—lung cancer-specific vs. extrapolated models

S. No	Nanocarrier system	Therapeutic strategy	Cancer model used	Lung cancer relevance	Key insight	Ref
1	UCMS Pep-aPDL1 nanocarrier	PD-L1 inhibition + peptide vaccine	Lewis lung carcinoma (mouse)	NSCLC-specific	Enhanced T-cell infiltration and tumour suppression	[79]
2	HA-PEI miR-125b nanoparticles	Macrophage reprogramming	EL4 lymphoma model	Non-lung (Extrapolated)	Demonstrates immune modulation but not lung-specific	[80]
3	ccc	Combination chemo-immunotherapy	Lewis lung cancer model	NSCLC-specific	Improved tumour targeting and immune activation	[81]
4	IO-FuDex3 nano-biohybrid system	PD-L1 + T-cell activation	4T1 breast cancer metastasis model	Non-lung (Extrapolated)	Suggests reduced toxicity but lacks lung specificity	[82]
5	AuPG nanoparticles	PD-1 combination therapy	General tumor models	Extrapolated	Promotes macrophage polarization and T-cell response	[83]
6	SGT-53 nanoparticle	TP53 gene delivery + PD-1 therapy	Clinical + lung cancer models	NSCLC-relevant	Demonstrates translational potential	[84]
7	Redox-sensitive liposomes (PD-L1 siRNA)	Gene silencing immunotherapy	Lung cancer models	NSCLC-specific	Reduced checkpoint expression and toxicity	[85]
8	MMP-responsive dendrimers	Controlled ICI delivery	Lung cancer mouse model	NSCLC-specific	Improved tumor penetration	[86]
9	Hypoxia-responsive nanoparticles	Targeted drug release	Mixed tumor models	Partially extrapolated	Needs lung-specific validation	[87]

### Combination strategies in lung cancer treatment

Lung cancer is among the most perilous malignancies, particularly NSCLC comprising around 85% of cases. ICIs have transformed lung cancer treatment through their PD-1 and PD-L1 inhibitors which restore the immune system’s ability to detect and destroy tumor cells. The immunosuppressive properties of the tumor microenvironment together with natural or acquired resistance mechanisms make ICIs ineffective for certain patients who show treatment resistance to the drugs [88]. The combination of immune checkpoint inhibitors and chemotherapy has developed into a successful method which improves treatment outcomes through their combined effects. Chemotherapy induces immunogenic cell death while boosting antigen presentation and reducing TME immunosuppressive cells, which creates ICIs sensitivity in tumors. The therapeutic benefits of systemic chemotherapy are restricted because it causes substantial toxicity and effects that extend beyond its intended targets. Nanomedicine-based drug delivery systems provide an advanced approach which improves the pharmacokinetics and tumor targeting capabilities of immune checkpoint inhibitors and chemotherapeutic drugs while enabling their controlled release.

Researchers developed nanocarriers which include liposomes polymeric nanoparticles dendrimers and lipid-based nanostructures to deliver immune checkpoint inhibitors together with chemotherapeutic agents more precisely. The nanoplatforms protect therapeutic compounds from rapid degradation while providing extended circulation time and enabling therapeutic compounds to accumulate in tumor tissues through enhanced permeability and retention EPR contact. When used with PD-1 inhibitors pegylated liposomal doxorubicin formulations show better tumor penetration and reduced overall body side effects [89]. Polymeric nanoparticles containing both immune checkpoint inhibitors and chemotherapeutic drugs have been engineered for sequential drug release, ensuring that chemotherapy-induced immunogenic cell death boosts the effectiveness of immune checkpoint inhibition. Research indicates that nanoparticle-mediated co-delivery of cisplatin and anti-PD-L1 antibodies significantly enhances tumour shrinkage and extends longevity in lung cancer models relative to monotherapy [90]. A primary benefit of nanomedicine in ICI-chemotherapy combinations is its capacity to alter the tumour microenvironment to promote anti-tumour immunity. The TME in lung cancer is markedly immunosuppressive, identified by the presence of tumour-associated macrophages, myeloid-derived suppressor cells, and regulatory T cells, which all work together to hinder effective immune responses against tumours. One possible side effect of chemotherapy is an increase in the number of CTLs and a decrease in the number of immunosuppressive cells [91]. Nanoparticle-based drug delivery devices augment this impact by facilitating the selective release of chemotherapeutic agents inside the tumour, hence reducing harm to healthy tissues. The pH-responsive nanoparticles that contain both paclitaxel and anti-PD-1 antibodies demonstrate their ability to improve T-cell infiltration while decreasing immunosuppressive elements in lung cancer research. The combination of passive targeting with active targeting capabilities through nanomedicine shows that the field can achieve dedicated tumor-specific antigen recognition through its ligands and antibodies [92]. Researchers have developed nanoparticles which carry specific ligands that deliver chemotherapy drugs and immune checkpoint inhibitors to cancer cells that show EGFR and PD-L1 protein markers, thus improving targeted treatment results and treatment performance. The antibody-conjugated nanocarriers improve immune checkpoint inhibition by blocking the PD-1/PD-L1 pathways which operate in the tumor microenvironment. The treatment approach achieves better results through direct treatment focused on specific patient needs because ICI treatment shows multiple side effects which include pneumonitis and colitis [93]. The main advantage which results from using chemotherapy together with immune checkpoint inhibitors through nanomedicine lies in the development of drug delivery systems which activate based on external signals. The intelligent nanocarriers deliver their therapeutic payloads when they detect specific triggers which occur in the tumor microenvironment through pH levels and enzymatic activity and redox circumstances. pH-sensitive nanoparticles [94]. The system discharges its contents into the acidic tumour microenvironment which ensures that chemotherapeutic drugs and immune checkpoint inhibitors receive their necessary treatment in precisely required locations. Enzyme-responsive nanoparticles disintegrate when they encounter the tumour-associated enzymes MMPs which enables the precise delivery of therapeutic drugs to specific sites [95].

These sophisticated nanocarriers markedly augment medication bioavailability, bolster immunological response, and diminish systemic toxicity. Recent advancements have investigated the function of nanomedicine in integrating ICIs and chemotherapy with supplementary treatment modalities, including PTT and PDT [96]. Gold nanoparticles and carbon-based nanomaterials have been used for PTT, which produces localised heat using near-infrared (NIR) laser irradiation, causing tumour cells to die and heightened immune activation. When integrated with immune checkpoint inhibitors and chemotherapy, photo-thermal treatment enhances tumour shrinkage and lowers recurrence rates [97]. Likewise, PDT, which employs light-activated photosensitises to generate ROS, has been incorporated into nano-carrier- mediated delivery methods to enhance the efficacy of immune checkpoint inhibition. These multimodal strategies use the unique characteristics of nanomedicine to augment anti-tumour immunity while reducing treatment-associated adverse effects. Notwithstanding the considerable potential of nanomedicine-based combinations of immune checkpoint inhibitors and chemotherapy, several obstacles persist in the implementation of these strategies in clinical settings (Table 3).

**Table 3:** Nanomedicine-based Combination Strategies for Lung Cancer Treatment

S No.	Combination Strategy	Mechanism of Action	Advantages	Challenges	Ref.
1	ICIs + Chemotherapy	Chemotherapy increases antigen presentation, enhancing immune response	Synergistic effect, increased tumor regression	Increased toxicity, immune-related adverse events	[98]
2	ICIs + Targeted Therapy	Precision medicine approach targeting mutations (EGFR, KRAS, ALK)	Enhanced tumor response, fewer side effects	Development of resistance, high cost	[99]
3	ICIs + Nanoparticles	Targeted delivery of ICIs, reduced systemic toxicity	Improved bioavailability, reduced off-target effects	Stability and large-scale manufacturing issues	[100]
4	ICIs + Photothermal Therapy	Heat-based tumor destruction combined with immune activation	Localized tumor control, minimal side effects	Requires specialized equipment, potential thermal damage	[101]

The mass manufacture of nano-carriers with uniform quality and stability is a significant challenge, since variations in nanoparticle size, charge, and surface characteristics might affect medication delivery efficacy. The complete development of nanomedicine formulations requires precise control of their pharmacokinetics and bio-distribution to ensure that drugs reach intended targets while avoiding accumulation in non-target body areas. The regulatory approval procedures for nanomedicine-based combination medicines need comprehensive preclinical and clinical validation to establish safety and effectiveness [102]. The management of lung cancer remains challenging because the disease exhibits multiple forms while it develops resistance to treatment and its tumor microenvironment suppresses immune functions. The combination of immune checkpoint inhibitors with chemotherapy and targeted therapy and radiation treatment has produced combined benefits that enhance treatment outcomes through immune system control and increased tumor fighting capacity [103]. Synergistic therapy occurs when two or more treatment methods work together to create better treatment results than any single method can achieve. The simultaneous use of chemotherapy with PD-1/PD-L1 inhibitors induces immunogenic cell death which improves antigen presentation and strengthens immune response. Radiation treatment increases tumor mutational burden which makes tumors more responsive to immune checkpoint inhibitors while increasing T-cell tumor infiltration [104]. The application of this combinatorial approach has resulted in higher response rates and longer survival times for NSCLC patients. The way individuals respond to treatment shows significant differences which create a requirement for treatment approaches that depend on genetic testing, molecular analysis, and immunological studies. Precision medicine establishes personalised therapy by developing treatment plans that match the distinct characteristics of a patient’s tumour which improves treatment results and decreases side effects. The development of next-generation sequencing and liquid biopsy technologies enables continuous observation of tumour growth which allows doctors to adjust their treatment methods according to the current situation [105]. Patients with EGFR mutations benefit from targeted therapy with Osimertinib whereas people with high PD-L1 levels show better results through immune checkpoint inhibitors. The use of artificial intelligence and machine learning in cancer research has improved personalised medicine through the identification of biomarkers which predict immunotherapy response and combination treatment outcomes. The TME single-cell RNA sequencing shows that immune cell populations exhibit diversity which enables development of more precise immune-modulating methods. The field of nanomedicine has advanced personalized medicine through its ability to enhance drug delivery systems which protect patients from harmful effects while achieving better treatment results [106]. The drug carriers which use nanoparticles as their foundation deliver medical treatments in a controlled manner while responding to tumor-specific signals that include pH changes and enzymatic activity and redox state alterations. The development of personalized nanomedicine approaches uses patient-derived tumor organoids and nanocarrier-based immunotherapy to create treatment protocols which match specific tumor characteristics. The development of biomimetic nanoparticles which use exosomes derived from tumors enables the delivery of customized immune checkpoint blockade treatments which improve treatment results while minimizing undesired effects. The combination of multi-omics data with nanotechnology has resulted in the development of hybrid nanocarriers which can simultaneously inhibit multiple pathways that contribute to lung cancer development. The field of personalized treatment through synergistic methods has made progress yet drug resistance and high treatment costs and the requirement for extensive clinical testing of new biomarkers represent major obstacles that still need to be resolved [107].

### Recent advances in ROS-modulating and phototherapy-based nanomedicine

Recent advancements in nanomedicine have increasingly focused on the integration of PDT, PTT, and ROS-modulating nanoparticles to enhance the safety and efficacy of cancer immunotherapy. The delivery system which uses nanoparticles has demonstrated its ability to enhance photosensitizers through three specific advantages which include improved stability and solubility and targeted tumor accumulation and the capacity to produce localized ROS when activated by external light sources. The targeted activation process establishes boundaries which restrict systemic exposure while decreasing off-target toxicity when compared with standard treatment methods [108].

The development of ROS-modulating nanoparticles has become an effective method for controlling oxidative stress in tumor microenvironments. The treatment system enables simultaneous destruction of tumor cells while protecting nearby normal cells from excessive oxidative damage. Researchers have proven that nanotechnology-based PDT and PTT treatments work effectively when combined with immunotherapy to produce immunogenic cell death which boosts anti-tumor immunity and maintains localized treatment effects. The combination strategies lead to better treatment results while decreasing the occurrence of irAEs which solve a major problem that exists with current immunotherapy methods [109].

The current situation shows two positive developments but three problems exist which include light penetration issues and tumor hypoxia and extended safety testing requirements. The research on multifunctional nanocarriers which respond to multiple stimuli provides solutions to existing limitations while demonstrating how these advanced systems can improve safety and effectiveness in lung cancer therapies.

### Recent advances in nanomedicine for lung cancer immunotherapy

The recent research progress in nanomedicine research has enabled doctors to conduct lung cancer immunotherapy operations with enhanced accuracy and better safety outcomes. The new platforms developed through this research enable treatment optimization because they provide doctors with advanced systems that target specific tumor sites while decreasing unwanted side effects. The research team developed stimuli-responsive nanocarriers because they needed a method to deliver drugs with planned timing and designated body locations. The researchers designed pH-responsive nanoparticles which use the acidic environment of tumors to release drugs exclusively at tumor locations while protecting healthy tissue from drug contact. The redox-responsive systems use intracellular glutathione levels, which increase in tumor cells, to control drug delivery to specific locations. The researchers developed enzyme-based and hypoxia-sensitive nanocarriers which use tumor biological signals for actual tumor detection. The smart delivery systems produce better results because they successfully decrease off-target effects while increasing the treatment effectiveness of immunotherapeutic agents [110].

Researchers have shown particular interest in combination therapy which uses nanocarriers to deliver medications from multiple agents. The combination of chemotherapeutic drugs with immune checkpoint inhibitors through co-delivery systems has shown in preclinical lung cancer studies to produce enhanced anti-tumor results. The study found that using nanoparticles to deliver doxorubicin together with PD-1/PD-L1 inhibitors led to increased tumor immunogenicity and decreased systemic toxicity because of controlled drug release. Similarly, nanocarrier-based delivery of siRNA targeting immune checkpoint pathways enables selective modulation of the tumour immune microenvironment [111].

### Clinical translation and challenges

The clinical translation poses considerable challenges owing to concerns about stability, bio-distribution, large-scale production, and regulatory endorsement. A key problem is the pharmacokinetics and bio-distribution of nanocarriers, since their build-up in non-target organs including the liver and spleen may result in off-target toxicity. Moreover, guaranteeing the repeatability of nanoparticle production at a clinical scale while preserving batch-to-batch uniformity continues to pose a technological challenge [112]. The safety profile of nanomedicine raises concerns, since long-term toxicity, immunogenicity, and clearance processes remain inadequately defined in human research. Moreover, authorities such as the FDA and the EMA in the US and Europe, among others, have strict requirements for the clearance of nanomedicine therapy, necessitating comprehensive preclinical and clinical validation. The expense associated with the development and manufacture of nanoparticle-based pharmaceuticals is a significant constraint, since intricate synthesis methods and specialised facilities lead to elevated production prices [113]. Moreover, diversity in patients’ immunological responses to nanomedicine presents a barrier in standardising treatment protocols. Although preclinical studies have shown encouraging outcomes, the effective clinical use of nanomedicine-based immunotherapies needs further extensive clinical trials to validate effectiveness and safety across varied patient groups [114].

**Fig. 3:**
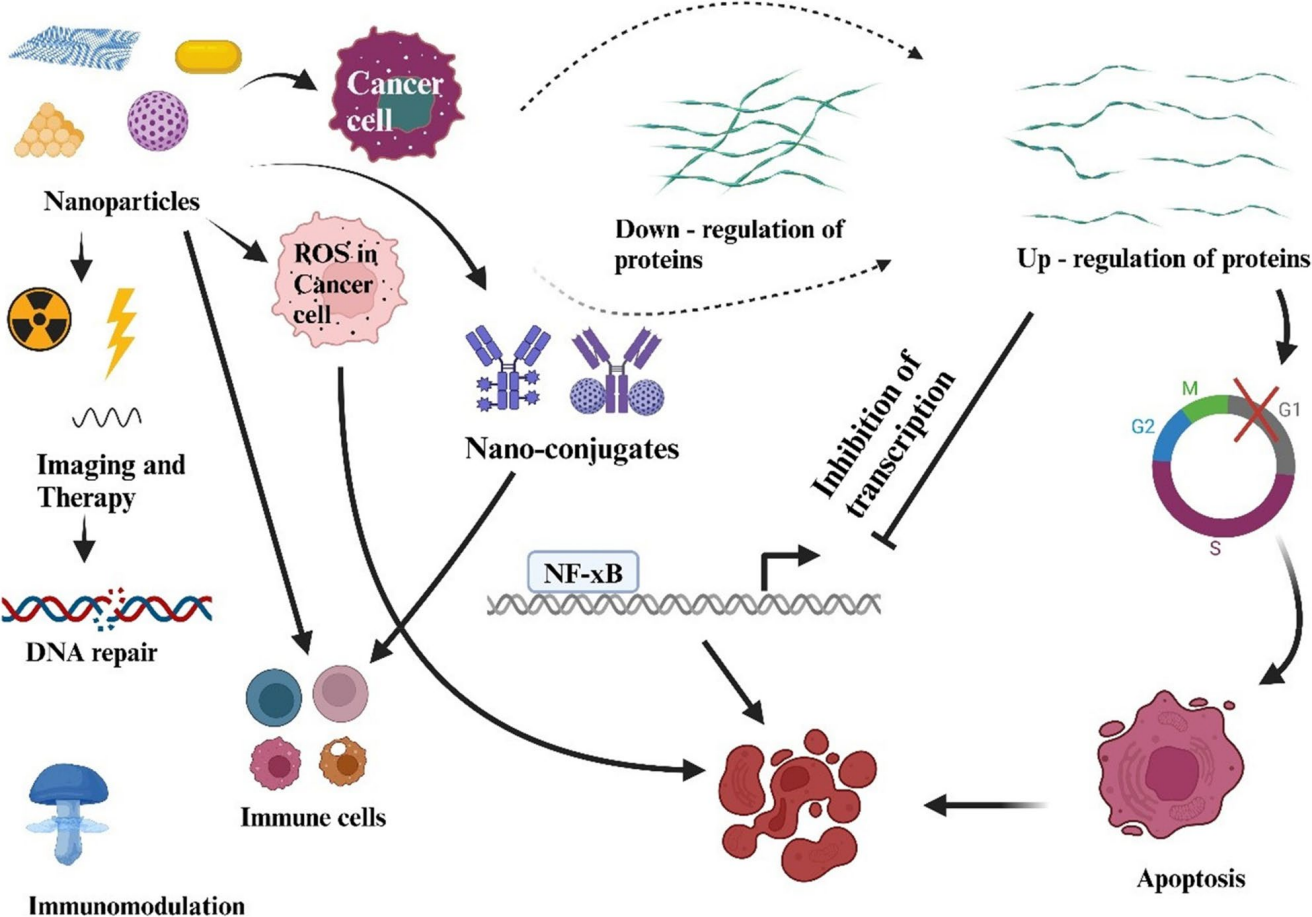
Clinical Translation Pathway for Nanomedicine in Lung Cancer [118]

The practical use of nanomedicine for lung cancer therapy is substantially impeded by issues of nanoparticle stability and biodistribution, which affect both therapeutic effectiveness and safety. The stability of nanoparticles is essential for preserving drug integrity, averting premature drug release, and facilitating regulated and prolonged drug delivery. Nevertheless, nanocarriers often encounter obstacles such as aggregation, degradation, and fast clearance from circulation, which constrains their efficacy [115] (Fig. 3). Variables include pH variations, enzyme activity, and interactions with plasma proteins might modify nanoparticle structure, resulting in diminished stability and unintentional drug release. Surface modifications using polyethylene glycol or lipid coatings have been implemented to improve nanoparticle stability and extend circulation duration [116]. Nonetheless, recurrent PEGylation may elicit immunological responses, resulting in accelerated blood clearance (ABC) and diminished therapeutic effectiveness. Bio distribution is a significant difficulty, since non-specific accumulation of nanoparticles in healthy organs including the liver, spleen, and kidneys may result in off-target damage [117].

The increased permeability and retention effect has been extensively used to augment nanoparticle accumulation at tumour locations; nevertheless, heterogeneity in tumour vasculature and the existence of biological barriers may limit this passive targeting strategy. Furthermore, fast elimination by the mononuclear phagocyte system (MPS) diminishes nanoparticle presence at the tumour location, requiring approaches to avoid immune detection [119]. Biomimetic nanocarriers, including cell membrane-coated nanoparticles, have potential in enhancing tumour targeting and minimising immune clearance. Active targeting techniques using ligand-functionalized or antibody-conjugated nanoparticles have improved tumour selectivity and reduced systemic toxicity [120]. Nevertheless, attaining equilibrium among extended circulation, effective tumour infiltration, and safe elimination continues to pose a difficulty. The incorporation of nanomedicine into lung cancer immunotherapy poses considerable regulatory and safety obstacles that must be resolved prior to broad clinical use. To ensure the safety, efficacy, and quality of nanomedicine treatments, regulatory agencies such as the FDA, EMA, and several international health organisations impose stringent requirements [121].

The primary regulatory challenge arises from the complex nature of nanomedicine formulations which contain various elements such as nanoparticles and medicinal drugs and targeting ligands and stabilizing coatings. The pharmacokinetic and bio-distribution and clearance properties of nanomedicines differ from those of conventional small-molecule drugs which require specialized assessment methods for their evaluation [122] (Table 4).

**Table 4:** Challenges in the Clinical Translation of Nanomedicine in Lung Cancer

S. No	Challenge	Description	Proposed Solutions	Ref
1	Stability and Bioavailability	Nanocarriers degrade or get cleared before reaching tumours	PEGylation, lipid coating, controlled-release strategies	[123]
2	Immunogenicity	Some nanoparticles trigger immune responses	Surface modification, use of biocompatible materials	[124]
3	Regulatory Approval	Complex testing procedures for new nano-based drugs	Standardized protocols, rigorous clinical trials	[125]
4	Large-Scale Manufacturing	High cost and complexity in producing nanoparticles	Scalable synthesis techniques, automation	[126]

The biological interactions of nanoparticles and their toxicity effects depend on their physicochemical properties which include their size and shape and surface charge and material composition. The regulatory agencies require complete characterisation studies to assess the stability and bioavailability and extended safety of nanomedicine products [127].

Researchers in nanomedicine address safety issues by investigating three main areas which include potential toxicity and immunological responses and the accumulation of substances in non-target organs over extended periods. The development of nano carriers seeks to improve drug delivery systems while reducing unwanted effects on the body but certain drug formulations can trigger unexpected immune system responses which lead to inflammatory disorders. Researchers found that PEG-coated nanoparticles which scientists use to enhance particle circulation time lead to the production of anti-PEG antibodies which result in faster particle elimination and reduced treatment efficiency [128]. The distribution of nanoparticles in living organisms shows unpredictable patterns which lead to their unintentional buildup in various body organs, particularly the liver, spleen and kidneys. The complete preclinical studies need to include both laboratory tests and animal tests because regulations mandate that all safety evaluations for nanomedicine products must be completed before clinical trials can start [129].

The solutions to these problems require standardized toxicity assessment methods which assess genotoxicity and immunotoxicity and conduct long-term biodistribution studies. The lack of standardized manufacturing methods and quality control standards for nanomedicine products represents a major regulatory challenge. Scientists need to ensure repeatable synthesis methods for nanoparticles because they rely on this process to produce therapeutic outcomes but current conditions require scientists to deal with variations in particle dimensions and electrical properties and medicinal content across different production batches [130]. The laws of Good Manufacturing Practice (GMP) require organizations to implement strict quality control measures which ensure that nanomedicine products maintain their uniformity and stability throughout the manufacturing process. The regulatory bodies emphasize the necessity of manufacturing methods which can be expanded to enable production while maintaining the complete integrity of nanoparticles and their medical functions without introducing any inconsistencies [131]. Researchers use advanced characterisation techniques which include dynamic light scattering and transmission electron microscopy and high-performance liquid chromatography to evaluate nanoparticle properties and their compliance with regulatory standards. The development of nanomedicine-based lung cancer treatments faces additional obstacles because clinical trials require extended periods to demonstrate safety and treatment effectiveness [132]. Phase I studies test nanomedicine formulations to determine their highest safe dosage which the body can tolerate and to assess their pharmacokinetic properties and potential side effects [133]. The studies of Phase II and Phase III research assess how effective therapies work and how patients respond to treatment and how safe the therapies remain over extended time periods with larger study groups. The development of nanomedicine faces major obstacles because clinical trials require high financial costs and long research timelines [134]. Regulatory authorities have implemented accelerated approval processes which include FDA Fast Track and Breakthrough Therapy designations to expedite the clinical development of potential nanomedicine treatments. The field of personalized medicine requires a solution to its regulatory challenges together with its ethical dilemmas that nanomedicine presents [135]. The success of nanomedicine treatments depends on three patient-specific factors which include genetic differences and immune system reactions and the diverse characteristics of their tumors. Ethical problems arise because resource-limited areas cannot provide equal access to advanced Nano therapeutics which require expensive manufacturing processes [136].

### Future prospective

The design of multi-stimuli-responsive nanocarriers that simultaneously respond to pH, redox conditions, hypoxia, or enzymatic activity represents another key advancement. The systems provide drug release which occurs at specific locations while maintaining precise control over the process, which leads to decreased immune system activation of unwanted effects. In parallel, AI-driven nanomedicine design is gaining attention for optimizing nanoparticle properties, predicting biodistribution, and personalizing treatment strategies based on patient-specific tumour and biomarker profiles. The combination of nano diagnostics with therapeutic platforms enables doctors to observe treatment progress and identify hazardous side effects which lead to modifications in medication amounts to create safer treatment methods. The development of ROS-modulating and phototherapy-based nanoplatforms will result in better control over tumors because these platforms work together with immune checkpoint inhibitors to reduce systemic adverse effects. The process of manufacturing products at a large scale and assessing safety over extended periods and developing regulatory standards alongside proving clinical effectiveness represents the main challenges that need to be addressed. Clinical trials that are well-designed require interdisciplinary collaboration to develop valid solutions which will connect preclinical research results with their clinical application. The new strategies which have emerged will create safer and more effective immunotherapy treatments which can be customized for lung cancer treatment.

## Conclusion

Lung cancer remains one of the hardest cancers to treat although immunotherapy has changed treatment methods. The use of ICIs faces limitations because they produce serious immune-related adverse effects. The field of nanomedicine shows potential to enhance drug delivery and drug absorption while minimizing harmful effects in pulmonary carcinoma treatment. The different nano transporters which include liposomes and polymeric nanoparticles and dendrimers have demonstrated strong capabilities to deliver targeted therapies while minimizing immune system-related side effects. The application of nanotechnology in immunotherapy has enhanced tumor penetration and immune response activation which results in improved patient results. The existing advancements face multiple obstacles which include challenges with nanoparticle stability and bio-distribution and large-scale manufacturing and regulatory clearance processes. The research needs to address these obstacles because they hinder the complete development of nanomedicine for lung cancer immunotherapy. The combination of biomarker-based patient selection methods with nanomedicine delivery systems will create future strategies that decrease immunotherapy toxicity while enhancing treatment accuracy.

## Data Availability

No datasets were generated or analysed during the current study.

## Abbreviations

APCs: Antigen-Presenting Cells
CTLA-4: Cytotoxic T-Lymphocyte-Associated Protein 4
CTLs: Cytotoxic T Lymphocytes
EGFR: Epidermal Growth Factor Receptor
FDA: Food and Drug Administration
HIF-1α: Hypoxia-Inducible Factor 1-alpha
IFN: Interferon
IL: Interleukin
irAEs: Immune-Related Adverse Events
KRAS: Kirsten Rat Sarcoma Viral Oncogene
LAG-3: Lymphocyte Activation Gene-3
MHC: Major Histocompatibility Complex
miRNA/miR: MicroRNA
MMPs: Matrix Metalloproteinases
NSCLC: Non-Small Cell Lung Cancer
PACIFIC: Programmed Death-Ligand 1 Antibody Consolidation After Chemoradiotherapy in Stage III NSCLC (clinical trial)
PD-1: Programmed Cell Death Protein 1
PD-L1: Programmed Death-Ligand 1
PDT: Photodynamic Therapy
PEG: Polyethylene Glycol
PEI: Polyethylenimine
PI3K: Phosphoinositide 3-Kinase
PTT: Photothermal Therapy
ROS: Reactive Oxygen Species
SCLC: Small Cell Lung Cancer
siRNA: Small Interfering RNA
TMB: Tumour Mutational Burden
TME: Tumour Microenvironment
TNF-α: Tumor Necrosis Factor-alpha
VEGF: Vascular Endothelial Growth Factor
VEGFR: Vascular Endothelial Growth Factor Receptor
